# New insights on the active degassing system of the Lipari–Vulcano complex (South Italy) inferred from Local Earthquake Tomography

**DOI:** 10.1038/s41598-022-21921-x

**Published:** 2022-11-07

**Authors:** C. Totaro, M. Aloisi, C. Ferlito, B. Orecchio, D. Presti, S. Scolaro

**Affiliations:** 1 Department of Mathematics, Computer Sciences, Physics, and Earth Sciences, University of Messina, Messina, Italy; 2 Istituto Nazionale di Geofisica e Vulcanologia, Sezione di Catania-Osservatorio Etneo, Catania, Italy; 3 Department of Biological, Geological and Environmental Sciences, University of Catania, Catania, Italy

**Keywords:** Geophysics, Seismology, Volcanology

## Abstract

Seismic tomography is a very powerful and effective approach to look at depths beneath volcanic systems thus helping to better understand their behaviour. The P-wave and S-wave velocity ratio, in particular, is a key parameter useful to discriminate the presence of gas, fluids and melts. We computed the first 3-D overall model of Vp, Vs and Vp/Vs for the Lipari–Vulcano complex, central sector of the Aeolian volcanic archipelago (southern Italy). The investigated area has been characterized in recent times by fumaroles, hydrothermal activity and active degassing. In particular, in the Vulcano Island, several episodes of anomalous increases of fumarole temperature and strong degassing have been recorded in the past decades and the last “crisis”, started in September 2021, is still ongoing. For tomographic inversion we collected ~ 4400 crustal earthquakes that occurred in the last thirty years and we used the LOcal TOmography Software LOTOS. The results clearly depicted two low Vp and Vp/Vs anomalies located up to ~ 8 km depths below Vulcano and the western offshore of Lipari, respectively. These anomalies can be associated to the large presence of gas and they furnish a first picture of the gas-filled volumes feeding the main degassing activity of the area.

## Introduction

**Figure 1:**
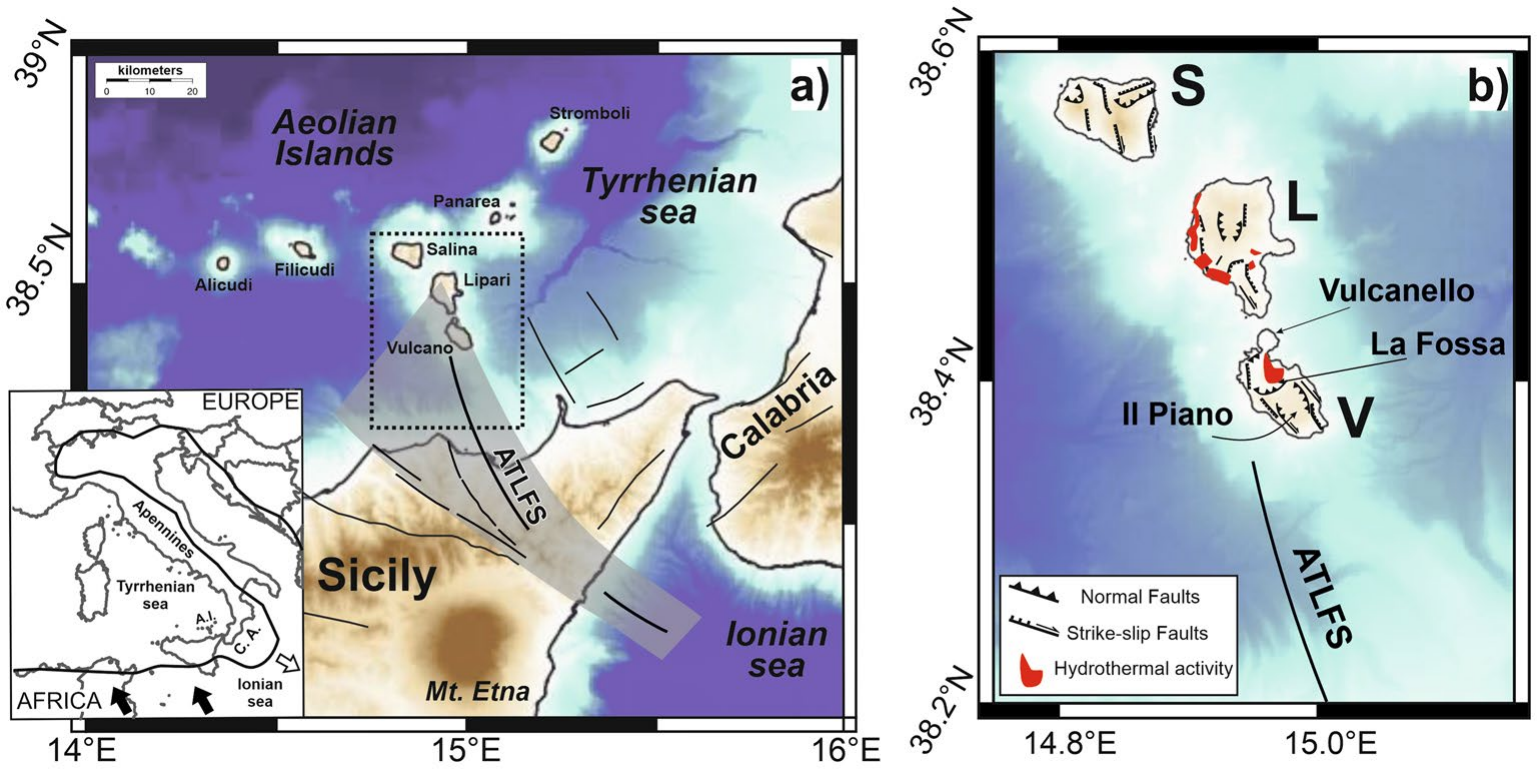
(**a**) Map view of the Southern Tyrrhenian region showing the area including northern Sicily, southern Calabria and the Aeolian Islands onto a simplified representation of the main fault systems. The gray belt highlights the Aeolian Tindari-Letojanni Fault System (ATLFS); the dashed rectangle encloses the area of main interest of this paper. In the inset, a geodynamic sketch of the central Mediterranean region: black arrows indicate the present-day motion of Africa with respect to Europe (according to Ref.^12^ and references therein); the white arrow indicates the orientation of the gravity-induced rollback of the Ionian subducting slab. C.A. and A.I. stand for Calabrian Arc and Aeolian Islands, respectively. Bathymetric data have been downloaded from https://portal.emodnet-bathymetry.eu/ and managed by using the software QGIS (version 3.10). (**b**) Enlarged view of the area of main interest of this paper (S, L, and V stand for Salina, Lipari, and Vulcano, respectively). The location of the hydrotermalized sources (red areas) and the main geological structures in Lipari, Vulcano, and Salina, redrawn from De Ritis et al.^24^ are reported.

Seismic tomography based on arrival times of the P- and S-waves from local earthquakes is a powerful tool actively used for studying volcanic systems. There are several examples of different volcanoes in the world, such as Merapi in Indonesia^1^, Mt. Etna in southern Italy^2,3^, Mt. Saint Helens in the United States^4^, among others, where tomography helped to successfully reconstruct the shallow-depth volcanic structure. Tomographic analyses have furnished, in particular, accurate pictures of the feeding systems and very precious constraints for modelling the volcanic processes also highlighting that each volcano has some peculiar features and appears to be unique. In this study we collected ~ 4400 earthquakes, recorded by a wide seismic network, to perform a tomographic inversion in the southern Tyrrhenian Sea region (South Italy), focusing on the volcanic archipelago of Aeolian Islands (Fig. 1). We used the software LOTOS^5^, already applied for several studies both in tectonic and volcanic frameworks^6–11^.

The Aeolian Archipelago is an arc-shaped structure located at the southern border of the Tyrrhenian Sea, in southern Italy, and it is composed of seven islands (Fig. 1). This region is mainly controlled by the NW–SE convergence between the African and European plates (e.g., Ref.^12^) and by the gravity-induced south-eastward rollback of the Ionian lithospheric slab subducting north-westward beneath the Tyrrhenian lithosphere^13,14^. The space–time evolution of the rollback process, affected by clear structural differentiation between the Calabrian Arc and the marginal tectonic units of Sicily and southern Apennines^15^, has been accommodated by the development of some shear zones bordering the forearc–backarc system (e.g., Refs.^16–19^). In this framework, the Aeolian volcanic ridge formed in Quaternary times along the Aeolian Tindari-Letojanni Fault System (ATLFS), a complex NNW–SSE to NW–SE-trending set of faults (Fig. 1) that may represent the easternmost and youngest of these tectonic accommodation structures^20–22^. The central sector of the Aeolian archipelago is crossed by a volcanic belt including the Lipari and Vulcano islands (Fig. 1), showing a NNW–SSE-elongation consistent with the orientation of the ATLFS. This belt is located above an about 20 km thick continental crust^13,23^, and has shown in the last decades large hydrothermal activity and active degassing^24–26^. In particular, the Vulcano Island has been characterized by several ‘‘crises’’ of alarming increases of fumarole temperature and strong degassing; the last of this crisis, started in September 2021, is still ongoing and is accurately monitored by the Italian National Institute of Geophysics and Volcanology (https://www.ct.ingv.it/index.php/monitoraggio-e-sorveglianza/prodotti-del-monitoraggio/comunicati-attivita-vulcanica).

In this framework we computed a new 3-D velocity model for the upper crust of the Lipari–Vulcano complex and we estimated for the first time in this sector, the distribution of the Vp/Vs ratio that allowed us to better characterize the structure beneath the Lipari–Vulcano complex and the related volcanic features.

## The Lipari–Vulcano complex

The Aeolian Islands and the associated volcanic seamounts are located between the Southern Tyrrhenian Sea back-arc and the Calabrian Arc orogenic belt (Fig. 1). Volcanism in the area started as the result of subduction of the Mesozoic Ionian slab beneath the Calabrian Arc (e.g., Ref.^27^ and reference therein) and it is related to a thermal uplift in a post-subduction extensional tectonic regime^28^. In this framework, the ATLFS is believed to have played a major role in the origin of the volcanic arc, highlighting its primary tectonic origin (e.g., Refs.^21,29,30^).

Looking at the central portion of the archipelago, Vulcano is a composite volcano characterized by different, NW–SE-elongated edifices, becoming gradually younger moving toward NW^31^. Below it, the presence of deflating magmatic sources and small magma reservoirs at different crustal levels has been hypotesized^25,27,32^. The island is dominated by NW–SE and NNW–SSE-striking fault systems (e.g., Fig. 1, Refs.^23,33^) that border the main morpho-tectonic depressions of Il Piano and La Fossa calderas (Fig. 1), therefore interpreted as pull-apart-type basins^23^. Vulcano displays a wide spectrum of eruptive-explosive activities due to the recurrent magma-water interaction^31^. The last eruptive phase occurred in 1888–1890 at La Fossa^34^ and it was characterized by dense lapilli-tuffs and obsidianaceous bread-crust bombs^24,27^. After this last event, volcanic activity has produced intense fumarolic emissions in correspondence with La Fossa and the north-eastern coast of the island^27^. Periodic variations in temperature and gas output caused by dynamics in the magmatic and hydrothermal systems are observed at the fumaroles located at the summit area of La Fossa^35^. Anomalous degassing periods have occurred in 1979–1981, 1985–1986, 1988–1991, 1996, 2004–2005, and 2009 (e.g., Refs.^32,36,37^). The last anomaly started in September 2021 and it is still ongoing (https://www.ct.ingv.it/index.php/monitoraggio-e-sorveglianza/prodotti-del-monitoraggio/comunicati-attivita-vulcanica). In particular, during the current unrest, diffuse soil CO_2_ degassing, high values of CO_2_ and SO_2_ fluxes at the summit fumaroles, high temperatures (about 350 °C) of the fumarolic gases and a variation of about 20 ppm in the areal dilatation of La Fossa, together with a centimetric uplift, have been observed (https://www.ct.ingv.it/index.php/monitoraggio-e-sorveglianza/prodotti-del-monitoraggio/comunicati-attivita-vulcanica). The anomalous degassing periods have been interpreted as a close interaction between tectonic dynamics, magmatic plumbing and shallow hydrothermal systems^37,38^.

Concerning the volcanism of Lipari Island (Fig. 1), the more recent activity (42 ka to 1220 AD) affected its southern and eastern sectors and was represented by pyroclastic fall and flow deposits, lava flows and domes. In particular, the last eruption, produced a rhyolitic pumice cone and obsidian lava^26^. Although this last volcanic eruption in Lipari occurred in 1220 AD, volcanic features such as hot springs and fumaroles observed at the surface indicate that volcanism is still active^24,39^. To date, hydrothermal activity characterizes the Lipari’s western sector and it is concentrated on faults and fractures aligned along a N–S direction (Fig. 1) that may control the location of the shallow magmatic reservoirs responsible for the more recent volcanism (< 55 kyr) at Lipari and Vulcano^23,26,40,41^.

## Results

**Figure 2:**
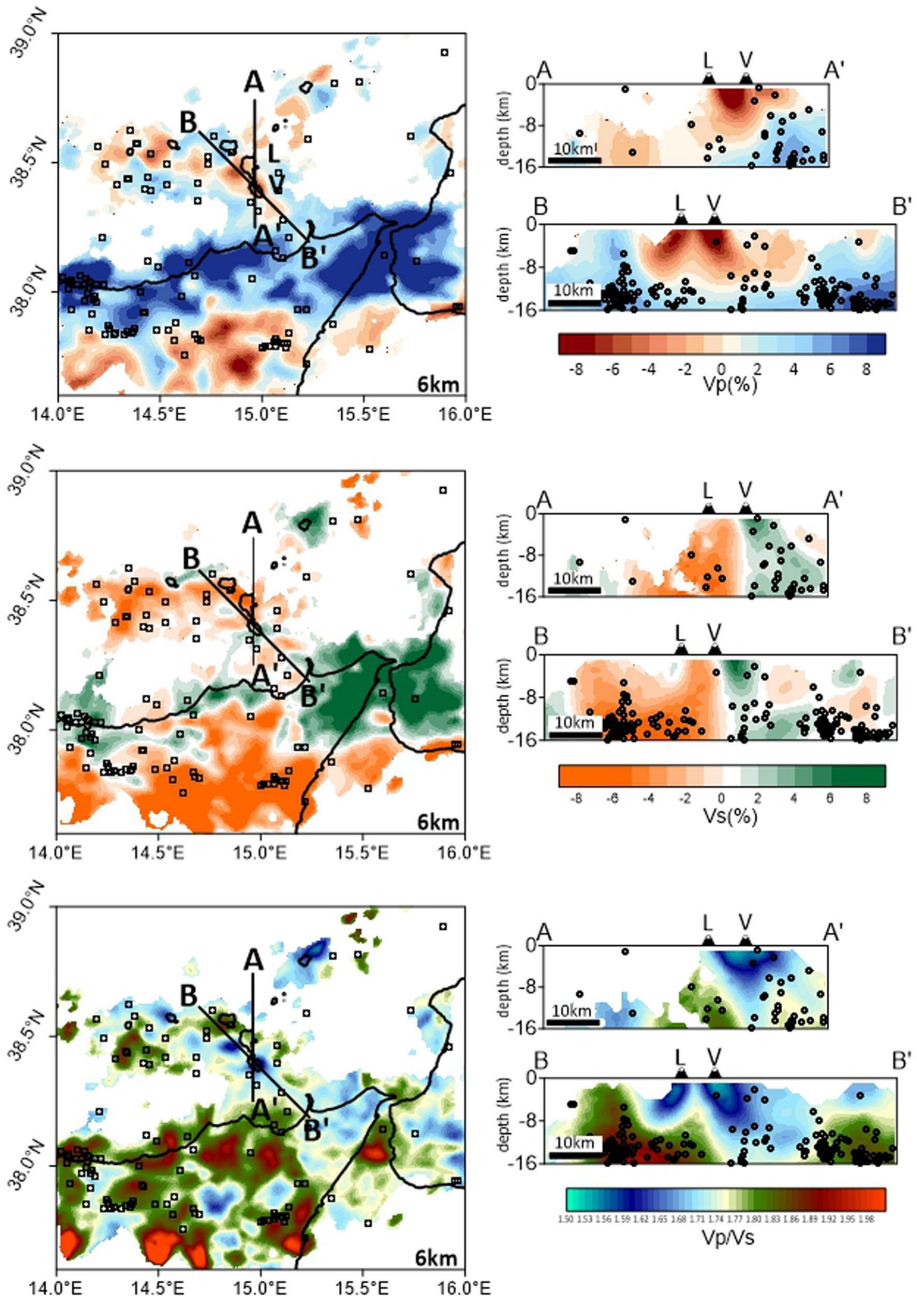
Tomographic results: map view of the 6 km depth level (left column) and two vertical sections (right column) cutting the Lipari–Vulcano complex (see the profiles AA′ and BB′ in the maps). The results obtained in areas with suitable data coverage are reported in terms of percentage variation of P- and S-wave velocity with respect to the optimal 1D reference model (top and middle) and in terms of Vp/Vs ratio (bottom), respectively. 3D earthquake locations (black circles) coming from the inversion are also shown: hypocenters in the focal depth range 5–7 km are reported on the 6 km depth level maps; events lying within ± 5 km from the vertical planes are displayed on the vertical sections. L and V indicate the location of Lipari and Vulcano islands. A more complete view of the tomographic results can be found in Fig. S1 (Supplementary Material).

The whole area of tomographic inversion is shown in Fig. 2. Taking into account the main features of the region, characterized by wide off-shore areas and consequent not optimal network coverage, we chose to investigate a larger volume around the area of main interest of our study (i.e., the Lipari–Volcano complex) in order to increase ray density and result resolution.

Tomographic results highlight the presence of a marked seismic velocity discontinuity between a relatively high P-wave velocity domain along the northern Sicilian coast and a low velocity one in mainland Sicily, to the south (Fig. 2 and Fig. S1). This change in the velocity pattern has been already observed by Totaro et al.^9^ and interpreted as a sudden transition from the low velocity body of imbricate thrust sheets and accretionary wedge of the Sicilian domain to the relatively high velocity of the Tyrrhenian continental crust of European origin. Below the central sector of the Aeolian Island archipelago, low Vp and low Vp/Vs patterns are also visible (Fig. 2 and Fig. S1). Velocity anomalies and earthquake distribution along two vertical sections reported in Fig. 2 highlight that the low Vp and low Vp/Vs anomalies are confined in the first 8–10 km of depth and that hypocenter distribution is mostly located within or close to the high Vp areas.

**Figure 3:**
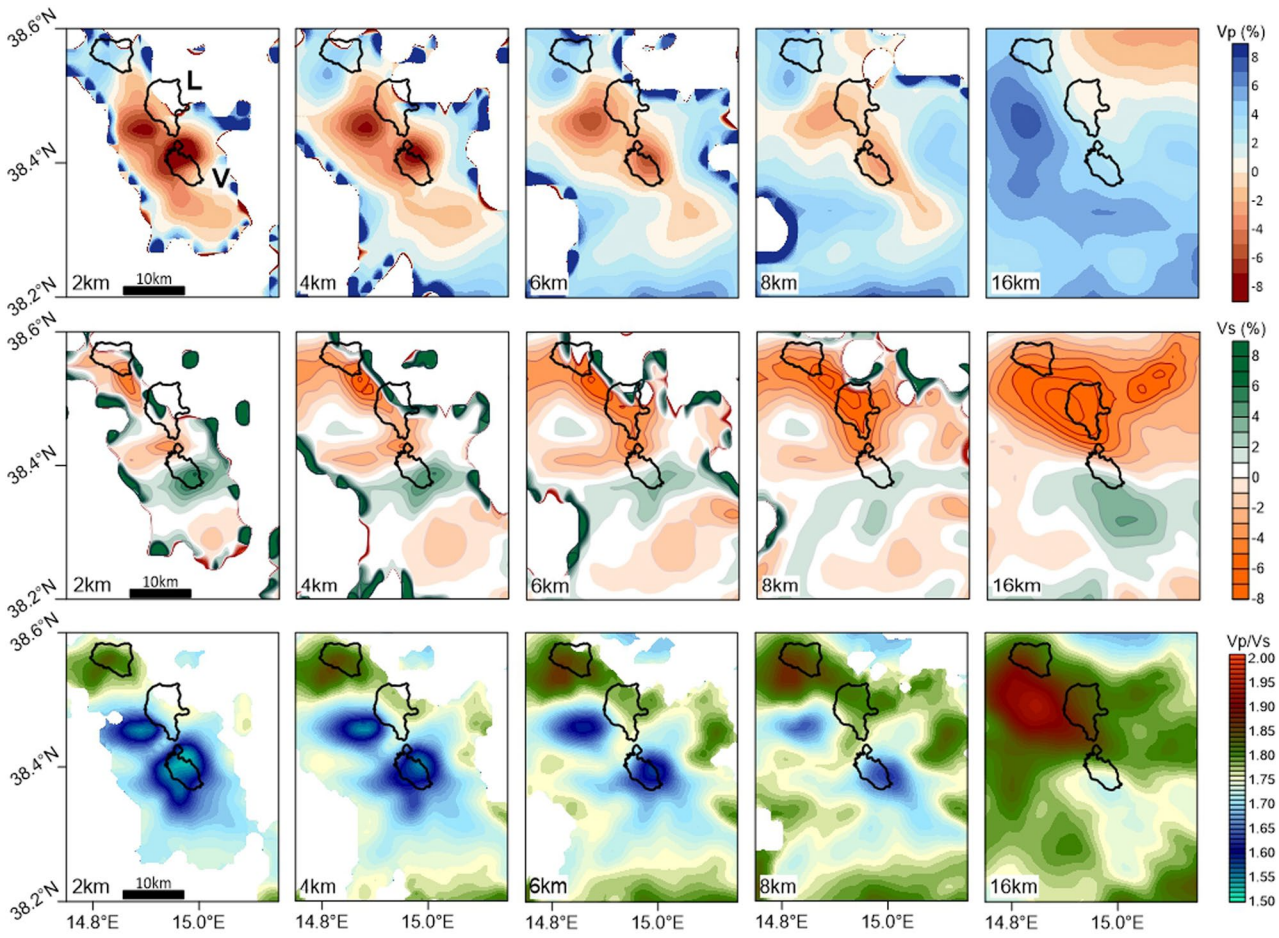
Enlarged view of the tomographic results in the area of the Lipari–Vulcano complex. The results obtained in areas with suitable data coverage are reported in terms of percentage variation of P- and S-wave velocity with respect to the optimal 1D reference model (top and middle) and in terms of Vp/Vs ratio (bottom), respectively. The number in the low-left corner indicates the b.s.l. depth in km.

Focusing on the area of strict interest of the present study (Fig. 3), the low P-wave velocity pattern clearly shows two distinct anomalies, reaching variations of − 8% and over, located in correspondence of Vulcano and in the western offshore of Lipari, respectively. In particular, at the shallowest level (2 km depth), the P-wave velocity beneath Vulcano shows a marked low velocity anomaly in the northern part of the island and relatively higher values in the southern part of it (Fig. 3), as also shown by Chiarabba et al.^42^. The two low Vp zones are well detectable up to 8–10 km depth while, at deeper depths, a high velocity pattern becomes the dominant feature (see Fig. 3 and Fig. S1).

The results obtained in terms of S-wave velocity indicate the presence of two main sectors: a high Vs zone in the southern part of Vulcano and a low velocity one immediately north of it (Fig. 3). This separation is quite evident in the whole depth-range of our investigation (see also vertical sections in Fig. 2 and Fig. S1).

Moreover, two patches of marked low Vp/Vs ratio can be clearly detected up to 8–10 km depth in correspondence of Vulcano and the western offshore of Lipari, respectively. These two anomalous zones well match with thelocation of the low Vp anomalies discussed above.

At deeper depth (16 km map in Fig. 3) high Vp/Vs values appear more evident in the north-western cornerof the area of our interest. This pattern, corresponding to a high-Vp and low-Vs area, is mainly centredin a volume marginal with respect to the Lipari–Vulcano complex, and needs to be deeply explored with further investigations properly focused on it and jointly evaluated with other geophysical data.

## Discussion

Seismic wave velocities provide relevant information about the structural features of volcanoes' feeding system. In particular, the Vp/Vs ratio distribution is often considered one of the most informative parameters in volcanic investigations, since, at the crustal level, it appears highly sensitive to the content of gaseous fluids and melts within rock bodies. As widely known, partial melted areas within the crust are characterized by low anomalies of both Vp and Vs, but also by high Vp/Vs ratio (e.g., Ref.^43^). Conversely, low Vp/Vs values are associated with a relevant presence of gas within the rock body (e.g., Ref.^44^). In fact, a medium saturated with gas behaves as a sponge and thus has a low compression modulus that causes low P-wave velocity values, whereas the decrease in S-wave velocity is not as substantial, determining a low Vp/Vs ratio^8,45^. Thus, it is widely believed that low Vp and low Vp/Vs anomalies are indicative of a gas-filled porous medium^46–48^. Nakajima et al.^49^ also highlighted that low Vp/Vs anomalies near the surface have been detected in geo- and hydro-thermally active areas. In addition, laboratory experiments provide evidence that Vp/Vs increases are associated with high temperatures and melt, whereas decreases may be linked to high gas content or supercritical fluids^50^.

On these grounds, it appears that joint evaluation of Vp and Vp/Vs is more meaningful for volcanic investigations than the S-wave velocity, appearing less sensitive to gas-content. The main evidence of our S-wave velocity model, that shows anomalies less marked than the Vp ones, is the separation between a high Vs domain below the southern part of the Vulcano island and a low velocity one in the northern sector of it (Figs. 2 and 3). This separation in the Vs patterns may reflect the volcanic history of Vulcano, being the younger volcanic edifices located in the north-western part of the island and the primordial volcanic apparatus (120–100 ka) in the southern one (e.g., Ref.^51^).

A joint evaluation of Vp and Vp/Vs results indicates a general pattern of quite low values of both Vp and Vp/Vs for the Lipari–Vulcano complex area by mainly showing two remarkable anomalies of low Vp and low Vp/Vs extending up to ca. 8–10 km depths and located below central-northern Vulcano and in the western offshore of Lipari, respectively (Fig. 3).

Even if we may not exclude the possible presence of minor magma reservoirs (e.g., Refs.^26,27,32^), possibly smaller than the resolution scale of our tomography, we remark that, according to the widely shared interpretation of Vp and Vp/Vs anomalies in volcanic frameworks, the velocity pattern we obtained is clearly representative of a high content of gas within the uppermost crust below the Lipari–Vulcano complex by mainly indicating two volumes of greater gas-concentration.

**Figure 4:**
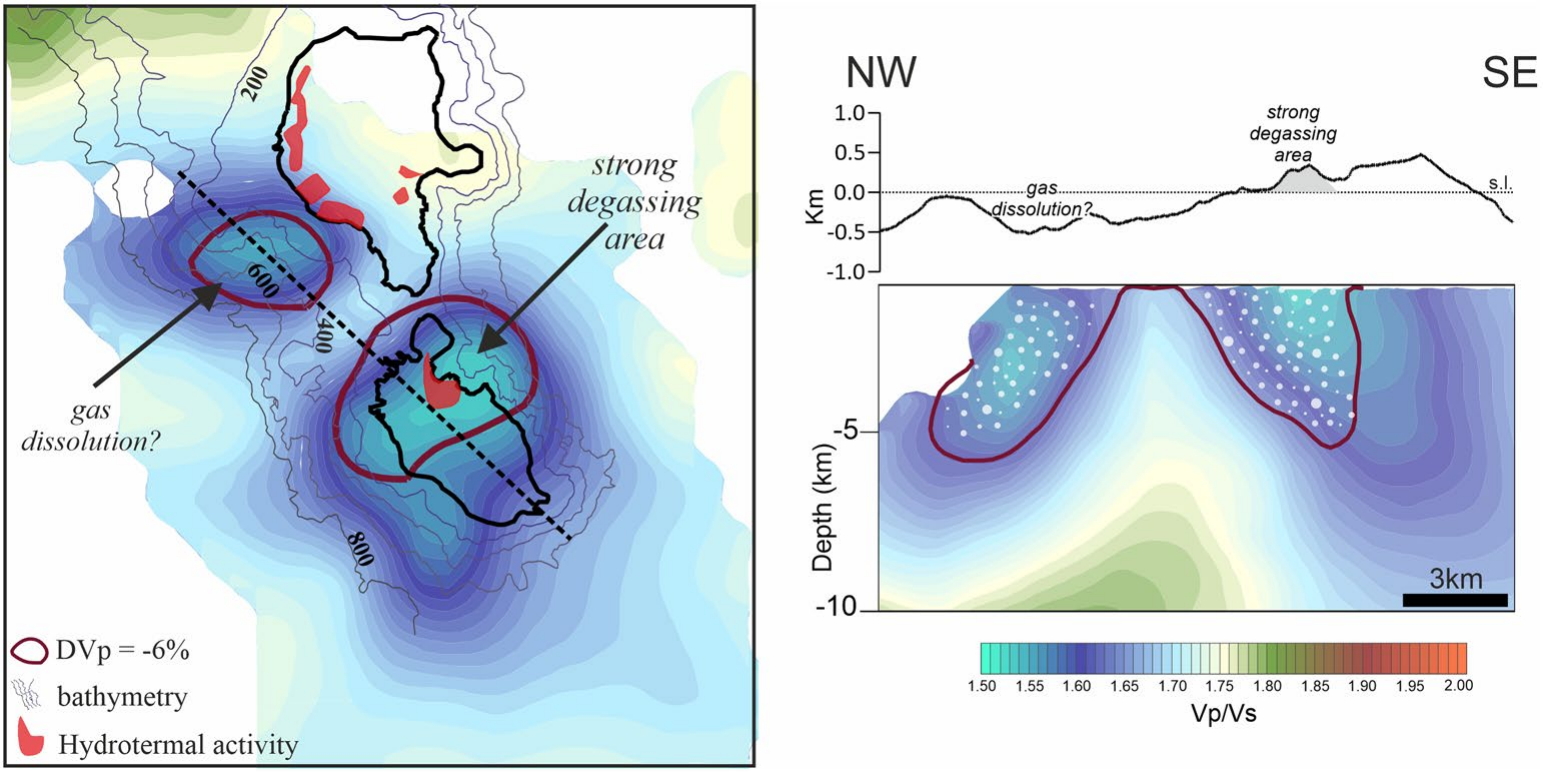
Sketch view of the main results of this study, highlighting the two anomalies of low Vp and low Vp/Vs we found beneath central-northern Vulcano and the western offshore of Lipari. In map we reported the tomographic results at 2 km depth in terms of Vp/Vs together with the Vp anomalies corresponding to a value of − 6% (purple lines). Isobaths of seafloor depth in correspondence with the main anomalies and sectors of documented hydrothermal activity^24^ are also shown (see legend). Along the vertical section cutting the two anomalies (see the profile in map) we analogously reported the Vp/Vs distribution and the contour lines of Vp-variation equal to − 6%. White dots represent the high gas content areas.

It has to be remarked that one of these anomalies associated to gas-filled rocks lies beneath the central-northern portion of Vulcano island, approximately in the La Fossa area (Fig. 1), and therefore in close correspondence with the sector usually characterized by intense fumarolic and hydrothermal activity (Fig. 4). Moreover, this low Vp and low Vp/Vs anomaly is centred just below the sector of the island mostly interested by the currently active crisis of alarming increases of fumarole temperature and strong degassing processes, started in September 2021 (https://www.ct.ingv.it/index.php/monitoraggio-e-sorveglianza/prodotti-del-monitoraggio/comunicati-attivita-vulcanica).

The other anomaly of low Vp and low Vp/Vs identified below the western Lipari off-shore suggests the presence of a second volume of relevant gas accumulation not previously detected in the literature. Possible degassing processes linked to this gas-filled volume are not directly observable due to the seafloor depth (ca. 600 m, Fig. 4): at this depth, in fact, the pressure of the water column would promote gas dissolution into marine water and therefore inhibit the gas bubbling observation. For this reason, the picture of uppermost crustal structures furnished by seismic tomography appears even more precious. In this scenario, we also note that the hydrothermal activity documented on the western shores of Lipari (Ref.^24^, among others) is located just near the boundary of the identified anomalous region and therefore it may represent the marginal shallow evidence related to the presence of gas in this sector.

Results obtained indicate that the upper crust of the Lipari–Vulcano complex, characterised by strong percentage variations of Vp and Vp/Vs, show low values of Vp (~ 3.8/4.0 km/s; Fig. S6) and Vp/Vs (~ 1.6). These values are quite different with respect to those computed for other active volcanoes in Sicily (Fig. 1). For example, Patane’ et al.^52^ deduced for Stromboli island high velocity bodies as a dominant feature in the Vp tomograms (6.5 ≤ Vp < 7.0 km/s) and relatively low Vs channels (Vs < 2 km/s; diameter of about 0.5 km) beneath the central craters, associated with high Vp/Vs zones (about 1.8 or more). The authors interpreted the low Vs and high Vp/Vs zones, similar to pipe-like structure, as highly fractured zones corresponding with shallow magma conduit that feeds the persistent present-day activity of Stromboli volcano; these zones could be associated with the presence of melt, predominant in the plumbing system. The strong difference, in terms of seismic velocities obtained for the Stromboli island and the Vulcano-Lipari complex, is clearly testified by their, at present, different volcanological behaviour (i.e. slow magmatic dynamics of the Lipari–Vulcano complex and persistent volcanic activity of Stromboli). Trying another possible comparison with the aim to better understand the seismic velocities here found, Mt. Etna represents an intermediate case, where a high Vp (~ 7 km/s) and Vp/Vs (~ 2) zone associated with fossil magma chambers is recognized, and zones characterised by low Vp (< 4 km/s) and low Vp/Vs (until 1.6) are associated to magmatic and volatiles pathways (e.g., Refs.^53,54^). These evidences together with quite similar absolute velocity values found in other volcanic regions (see e.g., Refs.^55,56^), also support the large presence of gas and a scarcity of melt component for the Lipari–Vulcano plumbing system.

The above described results lead us to consider that volcanic gas must be independent from molten rock. This idea, first conceptualized for Mount Etna^54,57,58^ and recently extended to Stromboli^59^, is based on the observation, for specific time-periods, of much more erupted gas with respect to the expected one according to the emitted volumes of volcanic rocks. In this new paradigm, the magma is described not as molten rock residing at a certain depth within the volcanic edifice or in the crust, but, on the contrary, as a flux of fluid continuously moving upwards in which two vertically distributed portions can be recognised (see Fig. 6 of Ref.^54^). At the bottom there is a solution made of ~ 70% in volume by a continuum gas phase (mostly H_2_O) in a supercritical state (density 360 kg/m^3^) and 30% in volume by silicatic components dissolved in it, named Water Melt Solution (WMS); the overall density of this layer is low (1140 kg/m^3^). At the top of the WMS, the continuous loss of gas, characterising all persistently active volcanoes, will leave a highly dense (2800 kg/m^3^) basaltic melt, defined as a Continuous Melt Phase (CMP). The CMP has a viscosity low enough to permit the continuous transit of the gas bubbles coming from the WMS degassing. The transition between the WMS and the CMP can be erratic within the plumbing system. In this scenario, the eruptions can be considered to occur due to a significant pressure increase within the WMS in the deep plumbing system, which cannot be kept confined by the weight of the overlying basaltic melt (CMP). However, as said above, the main features of volcanic activity of Mount Etna and Vulcano Island are quite different, so the feeding system model proposed for Mount Etna has to be properly adapted. In fact, the observation of the fumarolic activity and the low Vp and Vp/Vs found in Vulcano island, as described above, are compatible with this general model, but the absence of an open conduit at La Fossa caldera, together with the observed lack of strombolian activity and volcanic tremor, led us to suppose the absence of the CMP. Moreover, the WMS feeding the system must have a much higher fraction of gas than what has been hypothesized for Mount Etna. We might suppose that, in this specific case, the silicatic component within the gas flux is not relevant. Moreover, the scarcity of melt component within the WMS could be due to a general low temperature of the gas flux (T < 700 °C, below the melting temperature of basalts), as it can be noticed by measuring the temperature of the fumarolic field (https://www.ct.ingv.it/index.php/monitoraggio-e-sorveglianza/prodotti-del-monitoraggio/comunicati-attivita-vulcanica). In addition, we may note that if the relatively low-temperature gas flux originates low Vp/Vs values it might be plausible that a rise in gas temperature could produce silicatic melts, which in turn would modify the Vp/Vs. Thus, we can consider such velocity ratio as a valuable tool to detect significant changes in the physical state of the feeding system and therefore potentially useful as a long-range precursory signal.

## Conclusions

We estimated the first 3D overall model of Vp, Vs and Vp/Vs for the Lipari–Vulcano complex. The results of tomographic investigation indicate a relevant presence of gas-rich materials at shallow crustal depths. In detail, two main anomalies of low Vp and low Vp/Vs, related to gas presence, were observed beneath the central-northern sector of Vulcano and the western offshore of Lipari, respectively. The anomaly beneath Vulcano is located in close correspondence with La Fossa caldera area and with the sector where fumaroles, hydrothermal activity and active degassing are widely documented (Fig. 4; Refs.^24–26^, https://www.ct.ingv.it/index.php/monitoraggio-e-sorveglianza/prodotti-del-monitoraggio/comunicati-attivita-vulcanica). The observed anomaly therefore furnishes a picture of the spatial distribution of gas-filled volumes feeding the main degassing activity that is usually recorded in the Vulcano area and that also leads to some phases of alarming increase of the observed effects, like it is actually occurring.

Moreover, we also depicted a new, previously undetected, volume of strong gas-concentration beneath the western Lipari off-shore. Even if the two anomalies show almost the same intensity, no evidence of degassing activity is available for the latter one because of its location at sea depths where the relevant water column pressure inhibits the observation of possible degassing processes. Tomographic images, looking at depths beneath the volcanic region, have allowed us to obtain a first evidence of this region of anomalous gas concentration, needing to be further explored. Finally, an overall evaluation of our results, also compared with models previously proposed for other volcanic systems, strongly highlights the main role played by volcanic gas in the whole Lipari–Vulcano complex, thus furnishing invaluable constraints for improved modelling of the volcanic system and of its possible evolution.

## Methods

**Figure 5:**
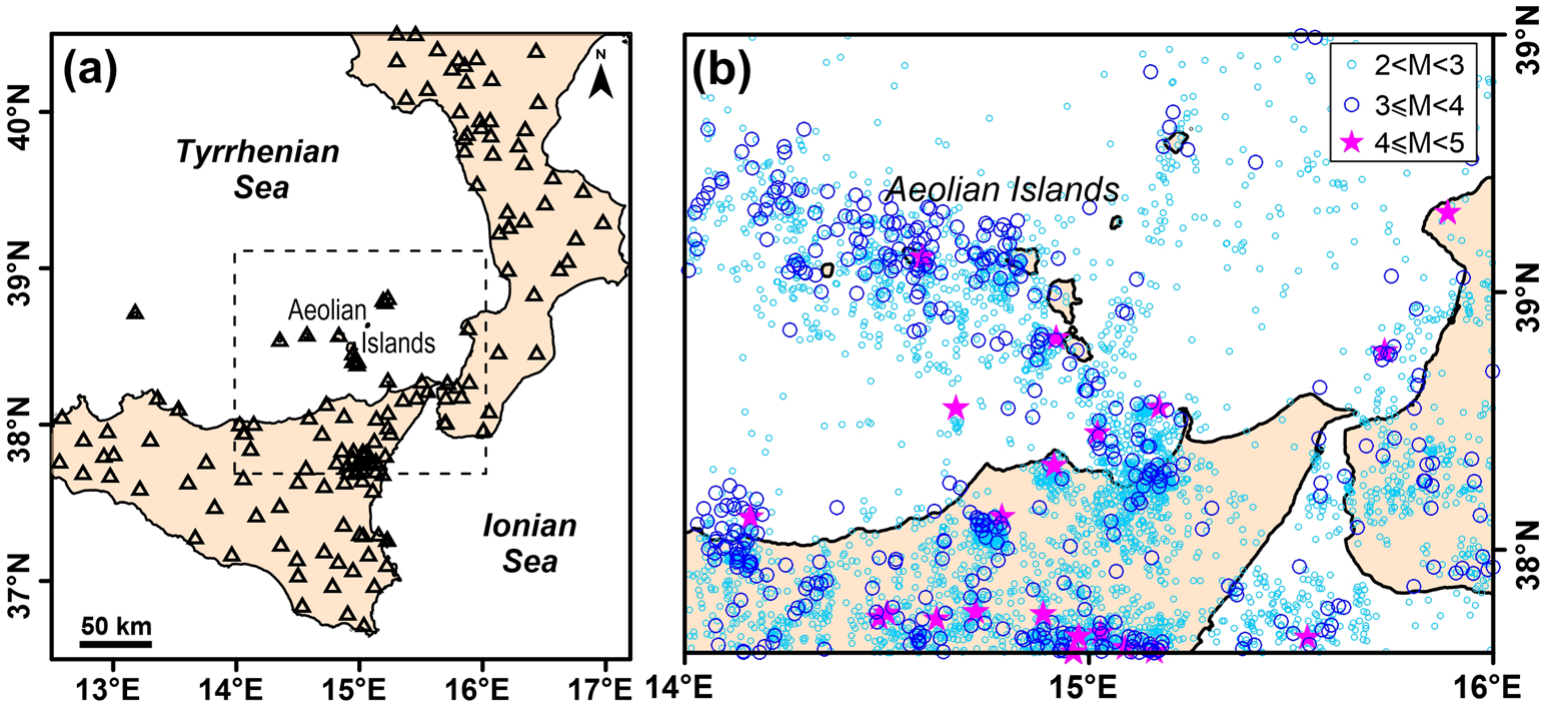
(**a**) Map of the seismic stations used in the present study (triangles). The dashed rectangular box indicates the tomographic inversion area. (**b**) Epicentral distribution of the starting inversion earthquake dataset. Events are reported with different symbols according to their magnitude (see legend).

To perform the tomographic inversion, we used data from local seismicity that occurred in the study region and were collected from the permanent and temporary networks managed by INGV (www.ingv.it). Time picks from seismic stations, including those located outside the study area, were used (Fig. 5a). We preliminarily selected earthquakes that have occurred between January 1990 and February 2021 in a wide region surrounding the Aeolian Islands (Fig. 5b) in order to obtain proper data distribution and ray coverage for the tomographic analysis. The obtained dataset consists of ~ 4400 crustal earthquakes with magnitude equal or greater than 2 that occurred in the depth range 0–40 km, with total number of P and S picks per event larger or equal 8. Following a selection procedure already successfully used in neighbouring sectors of the southern Tyrrhenian region (Refs.^6,9^) having comparable data quality, after locating the sources in the 1D starting model^9^, we excluded earthquakes with travel-time residuals greater than 1.5 s and 2 s for P- and S-readings, respectively. With this procedure we selected for the tomographic inversion a dataset of 3540 events having a minimum of 10 arrival times and average numbers of 15 P- and 8 S-wave picks (70% of events present more than 15 readings). This dataset corresponds to a total of 55,095 P- and 29,351 S-arrival times and the related ray tracing distribution shows that the volume of our interest, in the investigated depth range, is well sampled by the seismic paths both for P- and S-data (Fig. S2).

We modelled the 3D velocity structure by using the LOTOS code^5^. In this algorithm, the procedure starts with preliminary source locations based on the grid search method with the use of travel times computed in the 1D starting velocity model. Different 1D velocity models have been tested to find the most appropriate starting model, i.e. the one providing the maximum number of events and picks and the minimum arrival-time rms. Source locations in the 3D velocity model use 3D ray tracer based on the bending method, which ensures fast stable calculations of travel times of seismic rays between any two points in the study volume. Station corrections are also estimated during the inversion procedure. Velocity distribution parameterization is performed using nodes distributed within the study area according to the ray coverage. In this study, the volume was sampled with a regular horizontal step of 2 km while the vertical grid spacing, that is dependent on data density, cannot be smaller than 2 km, in our case. To reduce the effect of the grid geometry on the results, we performed the inversions for grids with four different orientations (i.e., 0°, 22°, 45°and 67°). The models computed for these grids were averaged in a regular mesh used as an updated 3D velocity model in the next iterations.

We obtained the 3D distributions of P- and S-velocity anomalies, while the Vp/Vs ratio was then computed by dividing the obtained values of absolute P- and S-velocities. Five iterations have been performed, leading to residual average deviations of 0.24 s and 0.35 s, and a consequent reduction of ∼ 38% and ∼ 40%, for P- and S-data, respectively, for the whole dataset. Detailed analysis on residuals carried out also for different subsets of events, particularly focusing on the area of interest of the present study, confirmed the relevant reduction observed for the whole dataset. The final station corrections are quite low especially in the region of main interest of this study (average values of the order of 0.01 and 0.02 for P- and S-waves, respectively) thus indicating that the tomographic results well reproduce the local velocity structure.

In order to carefully evaluate resolution and reliability of the obtained results, we performed a series of synthetic tests. In Fig. S3 is reported an example of checkerboard test performed by introducing periodic positive and negative velocity anomalies with amplitudes of ± 10% with respect to the 1D starting model and anomaly size of 9 km (comparable to the dimension of the anomalies detected in the present study). The synthetic travel times were computed for the same source-receiver pairs, as in the case of the observed data. The locations of real sources correspond to the solution obtained after five iterations of real data inversion. The synthetic data were perturbed with random noise having average deviations of 0.1 and 0.2 for the P- and S-data, respectively. P- and S anomalies are robustly reconstructed in most parts of the study area, in the investigated depth range (Fig. S3), highlighting the good quality and resolution of the obtained results.

In addition, several spike tests have also been performed by using different synthetic models reproducing anomalies fairly comparable with those detected by the tomographic inversion. In particular, we report in Figs. S4, and S5 the results obtained by defining two synthetic models consisting of (1) two low P-wave velocity anomalies of − 10%, no S-wave velocity anomalies, and consequently two low Vp/Vs anomalies, located in the 0–10 km depth-range beneath Vulcano and the western off-shore of Lipari, respectively (Fig. S4); (2) two low P- and S-wave velocity anomalies of − 10% (and consequently no anomalies in Vp/Vs), located in the 0–10 km depth-range in the same areas as above (Fig. S5). The results of these two tests showed that the correct values of the anomalies of Vp, Vs and Vp/Vs were retrieved within the initial anomaly limits, both in horizontal and vertical directions, indicating that the above discussed low velocity zones are located in a well resolved area, therefore they can be considered a reliable observation and not an artefact introduced by the inversion procedure.

Based on these tests, we evaluated that the size of patterns which can be resolved in the study area well corresponds to the size of the anomalies identified by the real tomographic inversion. Furthermore, these tests show that the inversion parameters, which were identical for real and synthetic data inversions, are adequate and provide optimal quality reconstructions.

## Supplementary Information

Supplementary Figures.

## Data Availability

Earthquake data are available on the INGV institutional website (http://www.ingv.it). The LOTOS code (version 12) can be downloaded from http://www.ivan-art.com/science/LOTOS. Figures reported in this paper have been managed by using the softwares QGIS (version 3.10), GMT (version 6), and CorelDraw (Technical Suite 2022). Data not available on these websites can be provided by the corresponding author on reasonable request.
