# Supplementary material for: New insights on the active degassing system of the Lipari–Vulcano complex (South Italy) inferred from Local Earthquake Tomography
Source: Sci Rep. 2022 Nov 7;12:18867. doi: 10.1038/s41598-022-21921-x (PMC9640725; doi:10.1038/s41598-022-21921-x)
Supplement: Supplementary file 1 — Supplementary Figures. [file 41598_2022_21921_MOESM1_ESM.docx]

**New insights on the active degassing system of the Lipari-Vulcano complex (South Italy) inferred from Local Earthquake Tomography**

C. Totaro^1,*^, M. Aloisi^2^, C. Ferlito^3^, B. Orecchio^1^, D. Presti^1^, S. Scolaro^1^

^1^Department of Mathematics, Computer Sciences, Physics, and Earth Sciences, University of Messina, Messina, Italy

^2^Istituto Nazionale di Geofisica e Vulcanologia, Sezione di Catania - Osservatorio Etneo, Catania, Italy

^3^Department of Biological, Geological and Environmental Sciences, University of Catania, Catania, Italy

*Corresponding Author: Cristina Totaro, ctotaro@unime.it

This supplementary document includes six figures (Figures S1-S6) reporting:

- a more complete and detailed view of the results of tomographic inversion (S1);
- the ray tracing of P- and S-waves (S2);
- the results of a checkerboard test (S3) ;
- the results of two spike tests with two anomalies performed to evaluate resolution and reliability of the 3D velocity structure (S4-S5);
- the absolute values of tomographic results for the area of main interest of the study (S6).

**
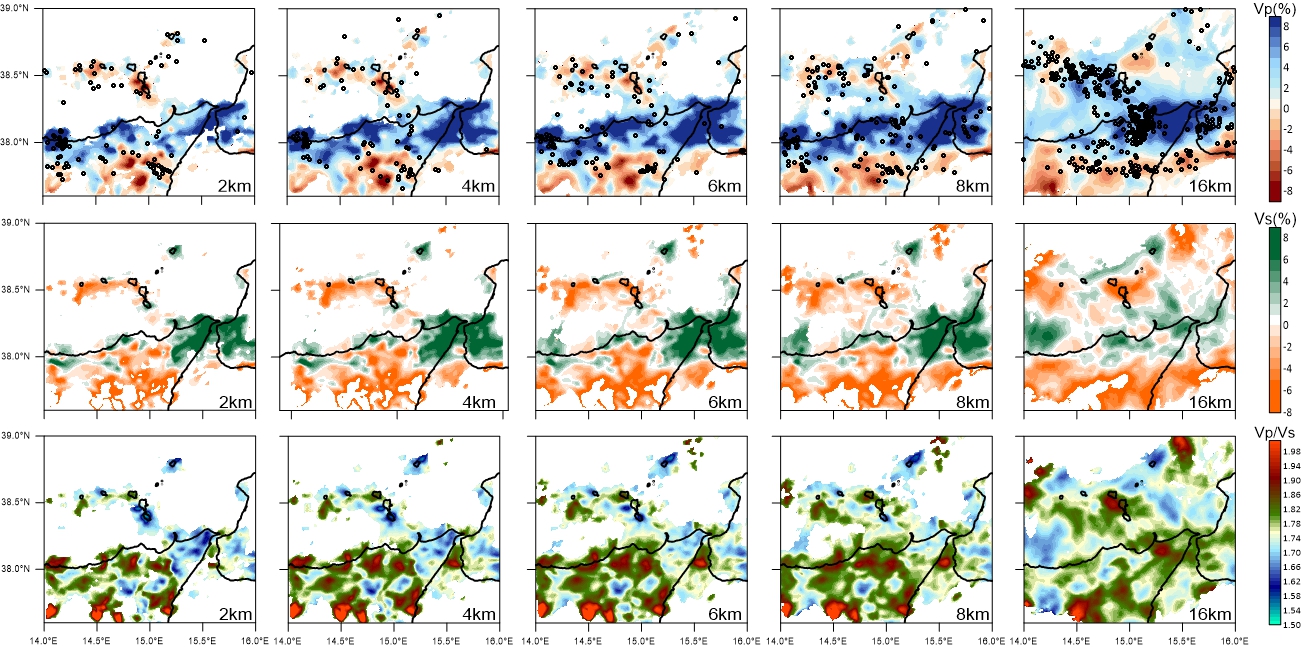
**

**Figure S1.** Tomographic model obtained in the present study reported in terms of percentage variation of P- and S-wave velocity with respect to the optimal 1D reference model (top and middle rows) and in terms of Vp/Vs ratio (bottom row), respectively. The number in the low-right corner indicates the b.s.l. depth in km.


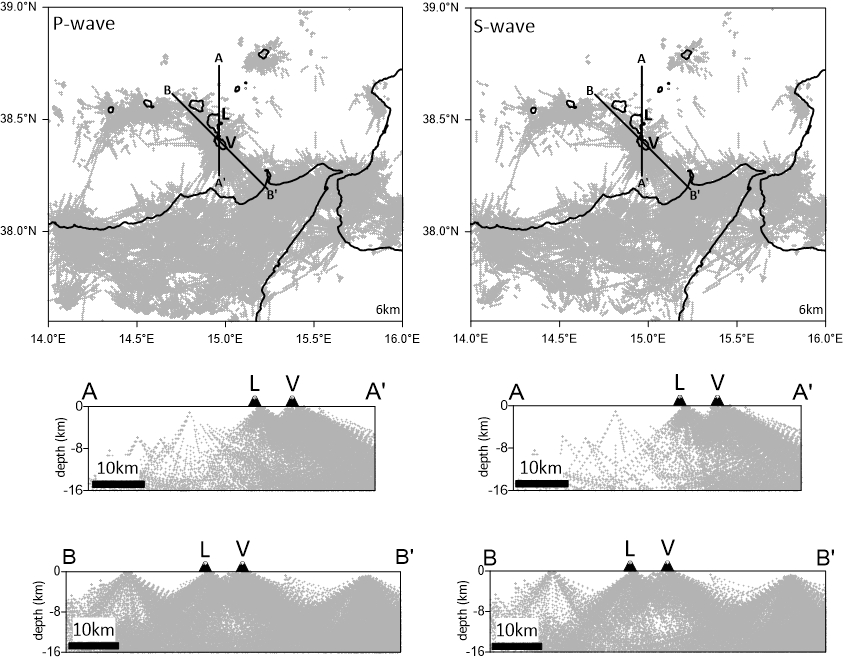


**Figure S2.** Horizontal (6 km depth; same level of Fig. 2) and vertical views (same profiles of Fig. 2) of the P-wave (left column) and S-wave (right column) ray tracings obtained for the inversion dataset in the optimal 1D reference model. Black lines in the maps correspond to the profiles along which vertical ray tracings are shown. L and V indicate the location of Lipari and Vulcano islands. The figure indicates the good ray-coverage in the area of main interest of the present study.


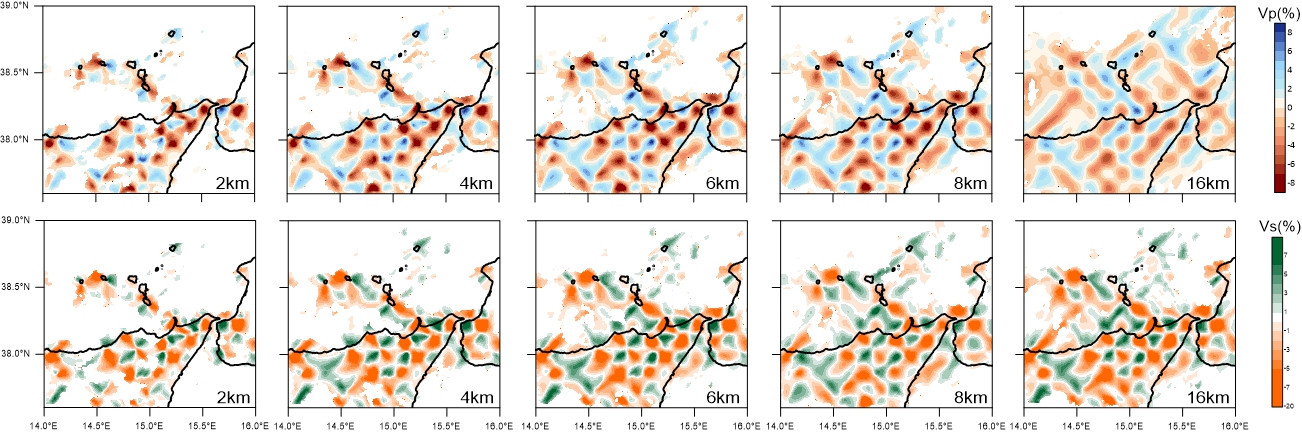


**Figure S3.** Results of a checkerboard test performed to assess the robustness of inversion and the spatial resolution of the tomographic model. Periodic positive and negative P- and S-wave velocity anomalies have been introduced with amplitudes of ±10% with respect to the 1D model, anomaly size of 9 km x 9 km. The number in the low-right corner indicates the b.s.l. depth in km.


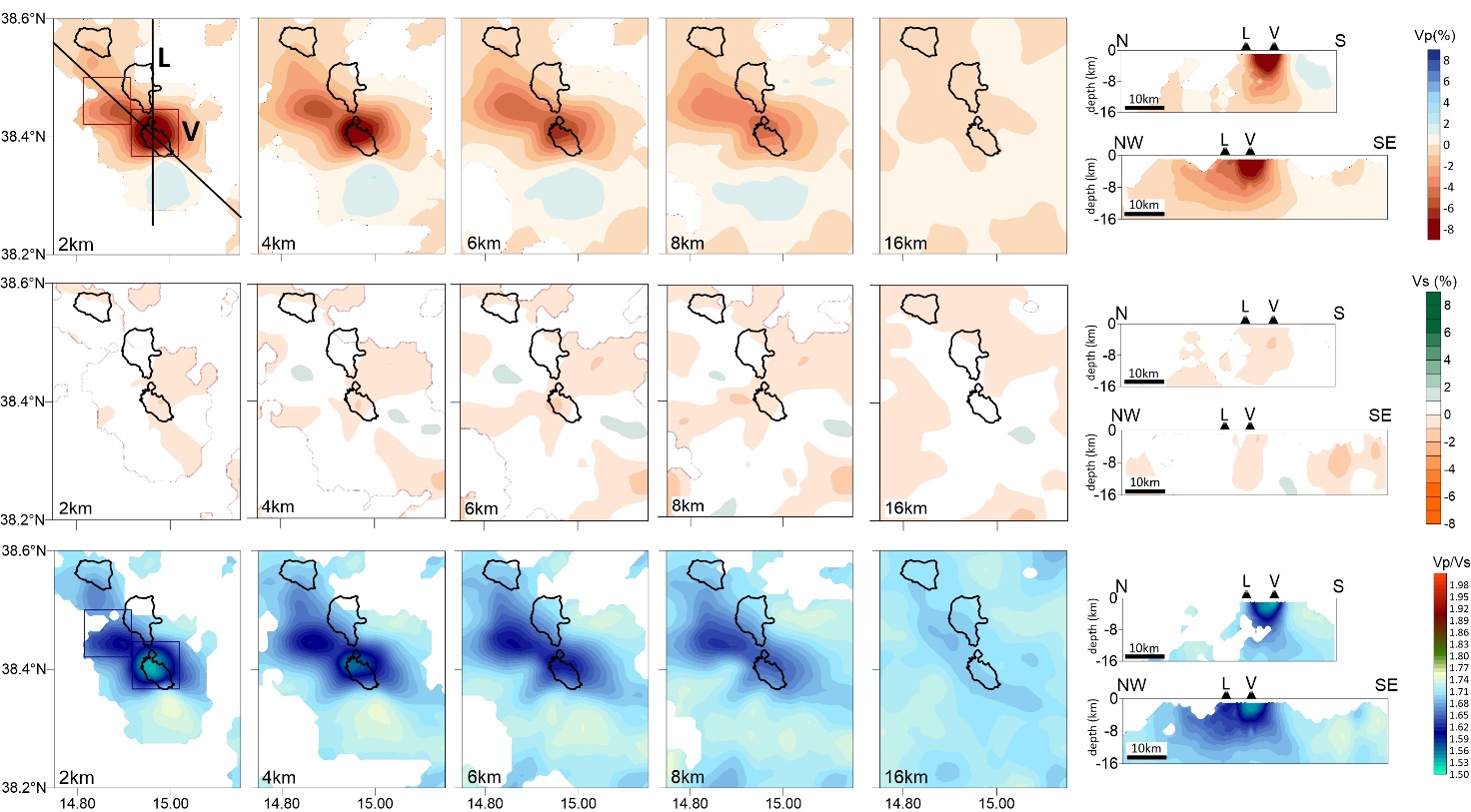


**Figure S4.** Results of a two-blocks synthetic test performed to assess the robustness of inversion and the spatial resolution of the tomographic model. The synthetic model structure, fairly reproducing the inversion results, is composed by two blocks (indicated as squares) characterized by low P-wave velocities of -10% and consequent low Vp/Vs values, located in the depth range 0-10km beneath Vulcano and the western offshore of Lipari, respectively. The number in the low-right corner indicates the b.s.l. depth in km. L and V indicate the location of Lipari and Vulcano islands. Results of the test along the two vertical profiles reported in the map at 2km depth are also shown.

**
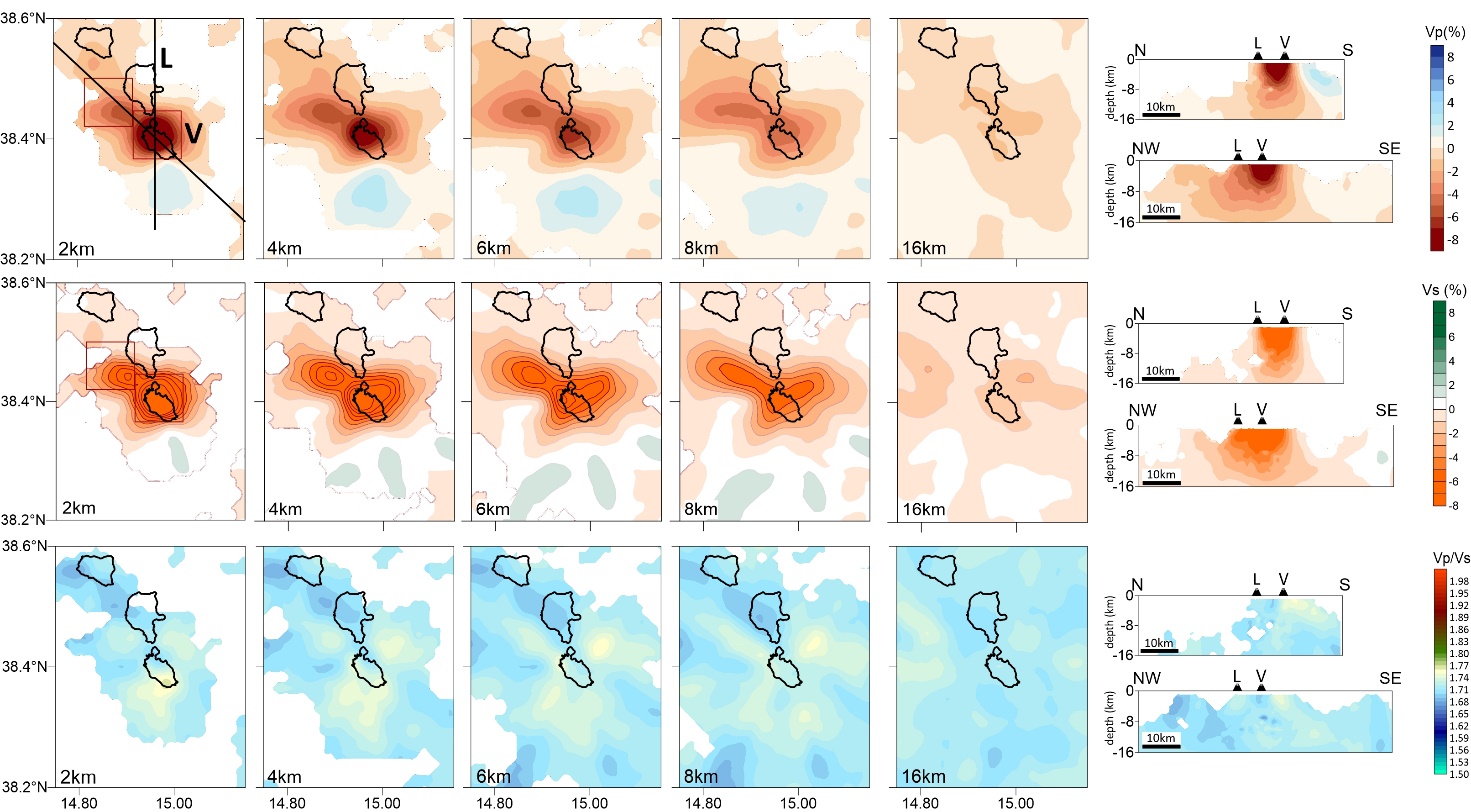
**

**Figure S5.** Results of a two-blocks synthetic test performed to assess the robustness of inversion and the spatial resolution of the tomographic model. The synthetic model structure is composed by two blocks (indicated as squares) characterized by low P- and S-wave velocities of -10% (and consequent no Vp/Vs anomalies), located in the depth range 0-10km beneath Vulcano and the western offshore of Lipari, respectively. The number in the low-right corner indicates the b.s.l. depth in km. L and V indicate the location of Lipari and Vulcano islands. Results of the test along the two vertical profiles reported in the map at 2km depth are also shown.


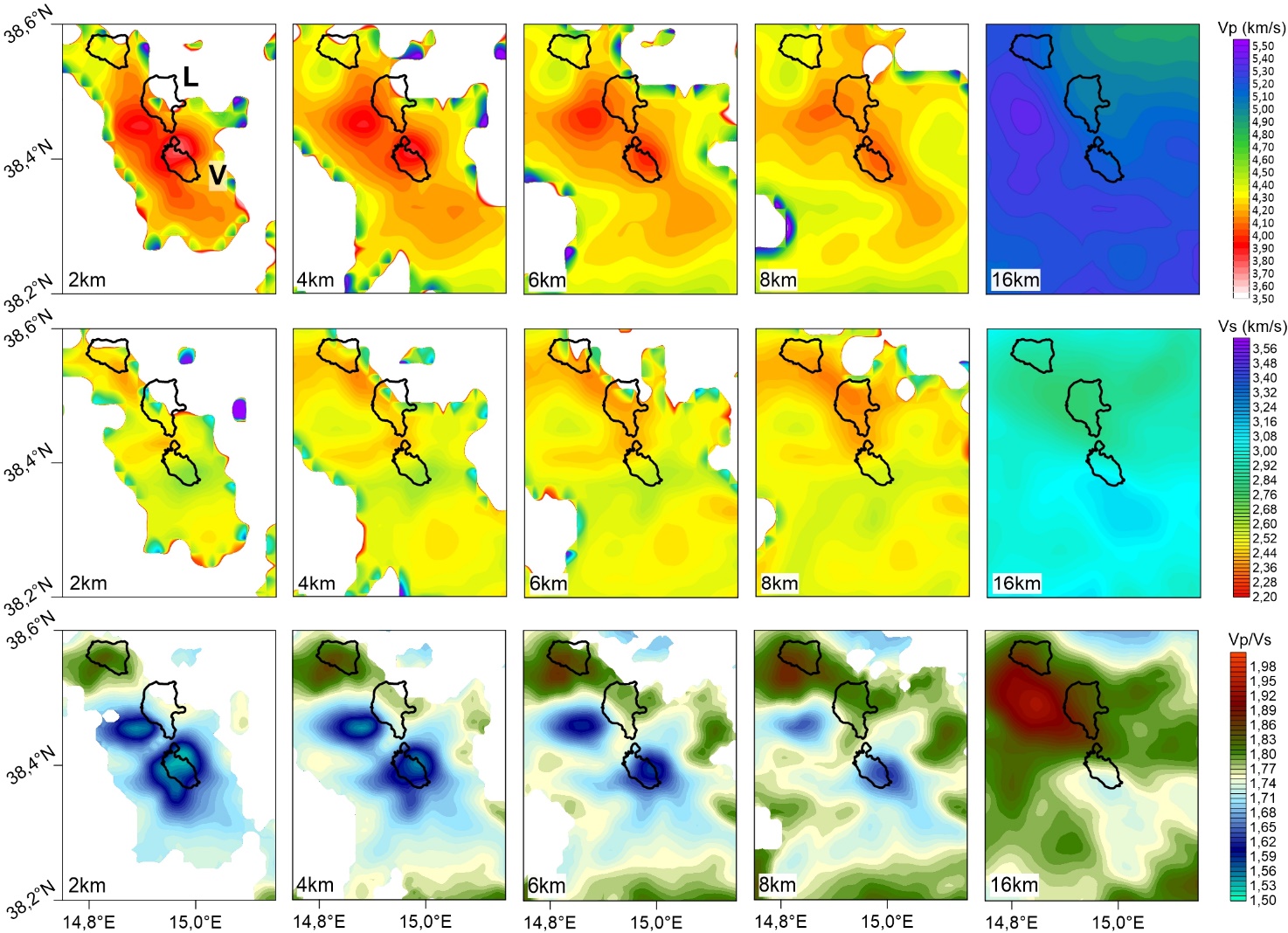


**Figure S6.** Tomographic results in the area of the Lipari-Vulcano complex reported in terms of absolute velocity values for P- and S-waves (top and middle) and Vp/Vs ratio (bottom), respectively. The number in the low-left corner indicates the b.s.l. depth in km.
